# Development and Comparative Evaluation of Ciprofloxacin Nanoemulsion-Loaded Bigels Prepared Using Different Ratios of Oleogel to Hydrogels

**DOI:** 10.3390/gels9070592

**Published:** 2023-07-23

**Authors:** Rania Hamed, Wala’a Abu Alata, Mohammad Abu-Sini, Dina H. Abulebdah, Alaa M. Hammad, Rafa Aburayya

**Affiliations:** Department of Pharmacy, Faculty of Pharmacy, Al-Zaytoonah University of Jordan, P.O. Box 130, Amman 11733, Jordan; walaataiseer92@hotmail.com (W.A.A.); mohammad.abusini@zuj.edu.jo (M.A.-S.); denaabulebdah@gmail.com (D.H.A.); alaa.hammad@zuj.edu.jo (A.M.H.); raffarayya99@gmail.com (R.A.)

**Keywords:** topical delivery, nanoemulsions, oleogel-in-hydrogel bigel, hydrogel-in-oleogel bigel, ciprofloxacin, rheology, antimicrobial activity

## Abstract

Nanoemulsions and bigels are biphasic delivery systems that can be used for topical applications. The aim of this study was to incorporate an oil-in-water ciprofloxacin hydrochloride nanoemulsion (CIP.HCl NE) into two types of bigels, Type I (oleogel (OL)-in-hydrogel (WH)) and Type II (WH-in-OL) to enhance drug penetration into skin and treat topical bacterial infections. Bigels were prepared at various ratios of OL and WH (1:1, 1:2, and 1:4). Initially, CIP.HCl NE was prepared and characterized in terms of droplet size, zeta potential, polydispersity index, morphology, and thermodynamic and chemical stability. Then CIP.HCl NE was dispersed into the OL or WH phase of the bigel. The primary physical stability studies showed that Type I bigels were physically stable, showing no phase separation. Whereas Type II bigels were physically unstable, hence excluded from the study. Type I bigels were subjected to microstructural, rheological, in vitro release, antimicrobial, and stability studies. The microscopic images showed a highly structured bigel network with nanoemulsion droplets dispersed within the bigel network. Additionally, bigels exhibited pseudoplastic flow and viscoelastic properties. A complete drug release was achieved after 4–5 h. The in vitro and ex vivo antimicrobial studies revealed that bigels exhibited antimicrobial activity against different bacterial strains. Moreover, stability studies showed that the rheological properties and physical and chemical stability varied based on the bigel composition over three months. Therefore, the physicochemical and rheological properties, drug release rate, and antimicrobial activity of Type I bigels could be modified by altering the OL to WH ratio and the phase in which the nanoemulsion dispersed in.

## 1. Introduction

Skin is the largest organ in the human body; it comprises ~16% of the total body weight and occupies an area of 1.8 m^2^ [1]. Using skin as a route of drug administration provides numerous advantages over other conventional administration routes, such as minimum invasiveness, ease of application, avoidance of first-pass effects, sustained drug release, enhanced patient adherence, and decreased systemic drug interactions [2]. Skin is made up of many layers, including the epidermis, dermis, and hypodermis [2]. The epidermis is the outer covering layer of the skin and is made up of keratinocytes structured into layers [3]. The stratum corneum (SC) is the outermost layer of the epidermis and creates a functional and physical barrier between the body and the environment [2]. Therefore, the development and optimization of topical drug delivery systems can overcome the major barrier of the skin (i.e., SC) [4]. Additionally, the permeation mechanism of the skin is affected by the physicochemical properties of the penetrating drug molecules and skin formulations [5]. For drug permeating the skin, the pKa, log P, molar mass (<500 Da), ionization/non-ionization fraction, diffusion coefficient of drug molecules, and solubility of the drug in the skin formulation play a fundamental role in the transportation of drug to the skin layers, whereas, the viscosity and hydrophilicity/lipophilicity of skin formulations and the presence of solvents, surfactants, and permeation enhancers in skin formulations are crucial in determining the efficiency of skin permeation [5].

Nanoemulsion (NE) is one of the lipid-based delivery nanosystems that have been employed to improve skin permeation of drugs. This biphasic nanosystem offers several advantages, such as nanosized drug-loaded droplets (<100 nm) that can easily pass through skin layers, low toxicity, non-irritancy, and enhanced skin permeation owing to the high concentration gradient of the drug and the presence of surfactants in the NE’s formulations [6,7]. Recently, our group has incorporated NEs into various gel-based formulations, including hydrogels [7], oleogels (or organogels) [8], and bigels [9], to extend their contact time with the skin, allowing more nanosized drug-loaded droplets to permeate the skin layers.

Bigels have recently been considered a distinctive class of gels for skin delivery [9,10]. Bigels are semisolid preparations composed of a combination of an aqueous-based gel (hydrogel, WH) and an oily-based gel (oleogel, OL), in equal or different proportions [11]. The rationale of preparing bigels was to reduce the defects of WH or OL separately, where WH restricts the ability of hydrophilic drugs to cross the skin barrier, whereas OL reduces patient compliance due to its oil residues and sticky-skin feeling. Additionally, bigels possess the characteristics of both WH and OL, including the ability to deliver lipophilic and hydrophilic drugs simultaneously to the skin, improving the hydration of skin layers, enhancing patient compliance by moisturizing, cooling, and emollient effects on the skin, and being easy to spread and wash when applied to the skin [9].

Bigels can be divided into three types, where the microarchitecture of the bigels changes based on the different ratios of OL and WH [10,12]. Oleogel-in-hydrogel (OL/WH) is the first type of bigel in which OL is dispersed within WH (continuous phase). Whereas hydrogel-in-oleogel (WH/OL) is the second type of bigel, in which the WH is dispersed within the OL (continuous phase). The third type is the bicontinuous bigel, where OL and WH are interpenetrating the two gel networks [10].

Ciprofloxacin HCl (CIP.HCl) is a fluoroquinolone antibiotic used to treat bacterial infections [13]. It belongs to a class IV drug with low solubility and low permeability [14]. It has a low minimum inhibitory concentration against Gram-negative and Gram-positive bacteria. Additionally, CIP.HCl is often used to treat a range of local bacterial infections, including ear, nose, eye, and skin infections. Moreover, it acts as a potential antibacterial for wound infections [15].

Therefore, the objective of this study was to prepare an oil-in-water (O/W) ciprofloxacin nanoemulsion (CIP.HCl NE) to enhance the penetration of the drug across the skin due to the small droplet size, the presence of surfactants that act as penetration enhancers, and the high drug-loading capacity due to the increased solubilization capacity of NE. Despite the many advantages, CIP.HCl NE lacks spreadability over skin due to its low viscosity, resulting in poor retention over the skin and hindering its clinical application. Thus, CIP.HCl NE was then incorporated into an oleogel (OL) or a hydrogel (WH) phase to prepare the two types of bigels, oleogel-in-hydrogel (OL/WH, Type I) and hydrogel-in-oleogel (WH/OL, Type II), at various proportions of OL and WH (1:1, 1:2, and 1:4). The incorporation of CIP.HCl NE into bigels improved the thermodynamic stability due to less Oswald ripening of the nanoemulsion, where the mobility of the nanoemulsion droplets in the oil phase decreased due to an increase in the consistency of the external aqueous phase. Additionally, CIP.HCl NE-loaded bigels increased retention time and enabled the formulation to release the drug in a controlled release dosage form for topical administration, benefiting the drugs with a short half-life. This may ultimately improve the clinical outcomes of CIP.HCl in treating bacterial infections of the skin. CIP.HCl NE-loaded bigels were investigated in terms of physicochemical and rheological properties, microstructure, in vitro release, antimicrobial activity, and stability. It is anticipated that the type of bigel and the ratio of OL to WH might significantly affect the aforementioned properties of bigels.

## 2. Results and Discussion

### 2.1. Preparation of Ciprofloxacin Nanoemulsion (CIP.HCl NE)

#### 2.1.1. Selection of CIP.HCl NE Components

**Figure 1:**
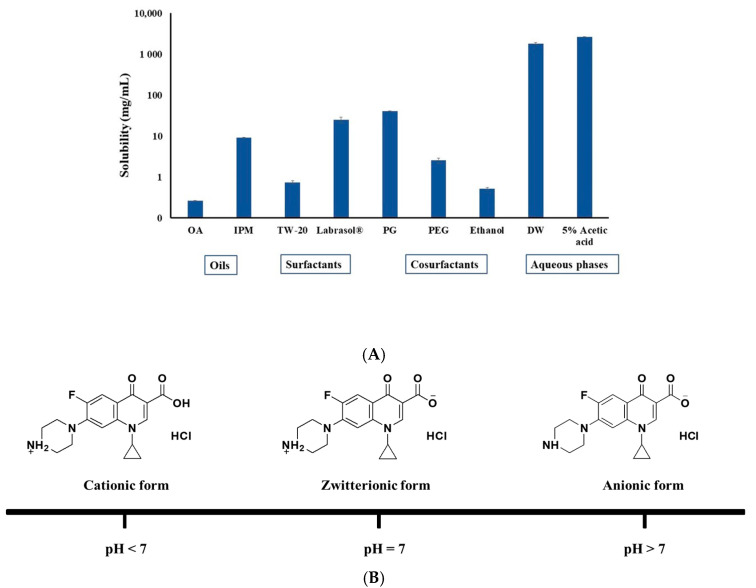

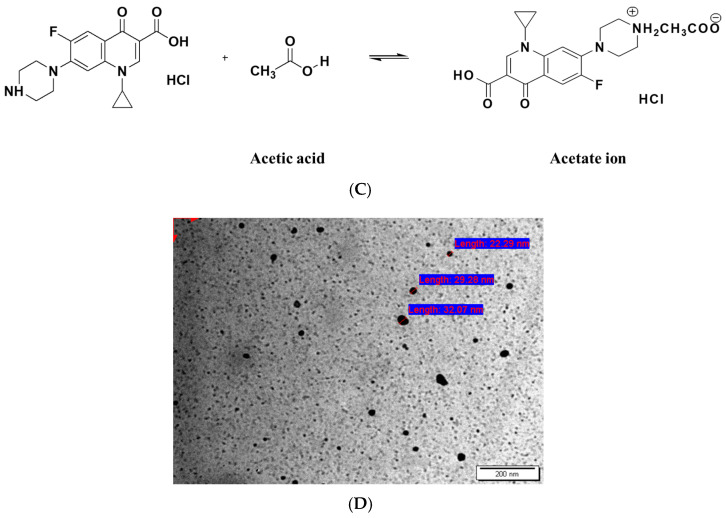
(**A**) Solubility of CIP.HCl in oils (OA and IPM), surfactants (TW-20 and Labrasol^®^), cosurfactants (PG, PEG, and ethanol), water, and 5% acetic acid. Data are presented as mean ± SD (*n* = 3), (**B**) CIP.HCl presents in different ionic forms based on the pH values (pH < 7, pH = 7, and pH > 7), (**C**) The interaction of CIP.HCl with 5% acetic acid, forming an acetate ion salt, and (**D**) a TEM image of CIP.HCl NE of spherical-shaped droplets.

The oil, surfactant, and cosurfactant of CIP.HCl NE were selected based on their maximum solubility of CIP.HCl and the clarity of CIP.HCl NE [16] (Figure 1A). Although the solubility in CIP.HCl in isopropyl myristate (IPM) was higher than that in oleic acid (OA) (9.2 ± 0.1 vs. 0.3 ± 0.02 mg/mL), OA was selected as the oil phase. This is because OA gives a homogeneous and clear mixture when mixed with the other nanoemulsion components. In addition, the solubility of CIP.HCl in Tween 20 (TW-20) was dramatically lower than in Labrasol^®^ (0.8 ± 0.1 vs. 25.1 ± 4.3 mg/mL). Therefore, Labrasol^®^ was chosen as a surfactant for the preparation of CIP.HCl NE. Moreover, the solubility of CIP.HCl in propylene glycol (PG) (40.9 ± 0.7 mg/mL) was higher than that in polyethylene glycol 400 (PEG 400) and ethanol (2.6 ± 0.3 and 0.5 ± 0.05 mg/mL, respectively). Therefore, PG was chosen as a cosurfactant. 

The solubility of CIP.HCl in distilled water (DW) and 5% acetic acid was extremely high at 1831.8 ± 122.5 and 2642.8 ± 10.7 mg/mL, respectively (Figure 1A). The high solubility of CIP.HCl in DW and 5% acetic acid is due to the presence of the drug in its water-soluble salt (hydrochloride), where CIP.HCl ionizes in water, forming ionic species [14]. CIP.HCl is a BCS class IV compound with amphoteric characteristics, indicating that CIP.HCl may exist in several microspecies in aqueous solution based on the pH [17] (Figure 1B). CIP.HCl is a zwitterion characterized by a biphasic solubility (i.e., highest solubility at low and high pH and poor solubility at pH ~ 7) [18,19]. The protonated piperazinyl moiety of CIP.HCl interacts with water, thus increasing the aqueous solubility of the drug [17]. Additionally, when the carboxylic group of acetic acid interacts with the piperazinyl moiety of CIP.HCl, an acetate ion salt is formed (Figure 1C). Because the solubility of CIP.HCl in 5% acetic acid was higher than that in water, the former was chosen as the aqueous phase for the CIP.HCl NE. Therefore, CIP.HCl NE was composed of OA (6.67% *v*/*v*), surfactant/cosurfactant of Labrasol^®^/PG (1:1) (60% *v*/*v*), and 5% acetic acid (33.3% *v*/*v*). The ratios of these components resulted in a clear and uniform CIP.HCl NE (Figure S1).

#### 2.1.2. Characterization of CIP.HCl NE

The CIP.HCl NE was assessed for particle size, polydispersity index (PDI), zeta potential (ζ-potential), morphology, and thermodynamic and physicochemical stability.

##### Droplet Size, PDI, and ζ-Potential

The droplet size, PDI, and ζ-potential of CIP.HCl NE were determined initially and after 7, 14, 21, and 28 days (Table 1). The initial droplet size of CIP.HCl NE was 17.27 ± 2.74 nm and attained sizes between 11.19 ± 0.11 and 22.88 ± 1.19 nm after 28 days. A statistical difference was found between the initial droplet size of CIP.HCl NE and those reported after 7 days (*p* < 0.01), 14 days (*p* < 0.01), and 21 days (*p* < 0.01). However, no statistical difference was found between the initial droplet size of CIP.HCl NE and those reported after 28 days (*p* > 0.01). Although there was a change in the droplet size of CIP.HCl NE over 28 days, the nanoemulsion droplets maintained their nanosized range of <100 nm [6]. The initial PDI of CIP.HCl NE was 0.21 ± 0.01, which was slightly increased after 28 days, ranging between 0.40 ± 0.02 and 0.49 ± 0.01. Nevertheless, these PDI values were <0.5, confirming the homogeneity and uniformity of the droplet size distribution [20]. The initial ζ-potential of the CIP.HCl NE droplets was 0.70 ± 0.01 mV, which increased to 1.57 ± 0.20 mV after 28 days. The relatively low ζ-potential is attributed to the non-ionic surfactant (Labrasol^®^), used at a relatively high concentration (30.0% *v*/*v*) [21]. Despite the fact that the ζ-potential of CIP.HCl NE is close to 0, the thermodynamic and chemical stability are attributed to the steric stabilization, where the thick layer of the non-ionic surfactant Labrasol^®^ with long side chains, surrounding the nanoemulsion droplets, provides a strong energy barrier to coalescence [22].

##### Morphology of CIP.HCl NE

The morphology of CIP.HCl NE, performed by transmission electron microscope (TEM), revealed that the droplets were uniformly dispersed, spherical in shape, distinct, and not aggregated, with a size ranged between 22.29 and 32.07 nm (Figure 1D).

#### 2.1.3. CIP.HCl Content

The CIP.HCl content in CIP.HCl NE was determined by HPLC, based on the linear equation (Y = 113653x + 6528.3, R^2^ = 0.9997) generated from a calibration curve of CIP.HCl in the mobile phase. The CIP.HCl content was 100.6 ± 2.9%, demonstrating that CIP.HCl was homogeneously distributed within the nanoemulsion. The amount of CIP.HCl was not determined in the internal (oil) phase. Due to the high solubility of CIP.HCl in the aqueous phase, it is anticipated that CIP.HCl will be preferentially deposited in the aqueous phase [23].

#### 2.1.4. Stability Studies of CIP.HCl NE

##### Thermodynamic Stability

CIP.HCl NE was physically stable, displaying no creaming, cracking, or phase separation after passing centrifugation and six heating/cooling (45/4 °C) cycles. However, during the freezing/thawing cycles, CIP.HCl NE crystallized at a very low temperature (−21 °C) (Figure S2). However, crystallization disappeared upon thawing at room temperature.

##### Chemical Stability

Stability tests of CIP.HCl NE were carried out at 2–8 °C, room temperature (22 °C), 30 °C/65% RH, and 40 °C/75% RH for three months. Initially, the CIP.HCl content in CIP.HCl NE was 100.6 ± 2.9%. The results showed that CIP.HCl NEs were chemically stable; no reduction in the CIP.HCl content was found in the stored samples after three months, ranging between 96.5 ± 4.2 and 105.6 ± 1.2% (Table 2).

### 2.2. Preparation and Physical Appearance of Bigels

Oleogelation is the process of entrapping liquid oils in a gel-like structure [24]. Ethyl cellulose (EC) was used as an oleogelator to prepare OL [25]. The OL was prepared by heating EC-containing OA at 140–150 °C, above the glass transition temperature of EC (around 130 °C) [26]. The EC-containing OA mixture was stirred constantly and cooled at lower temperatures. Upon cooling, the polymer strands formed a continuous network connected by hydrogen bonds. During the cooling phase, the liquid oil was physically trapped within the polymer strands, resulting in the formation of a gel-like structure [24]. WH was prepared by dispersing the hydrogelator Acrypol*^®^* 971P (CP) in an aqueous medium, which causes it to uncoil and expand. With the addition of trimethylamine (TEA), the carboxylic acid moieties neutralized, resulting in gelation. The three-dimensional network that is stiff and strong is related to the electrostatic repulsion between the polyelectric chains [27]. Bigels were formed when the hot mixture of OL and WH was cooled down to room temperature. Initially, the WH of pH 9–10 was mixed with the OL, forming a network within the bigel. Then, the pH of bigels was adjusted to 6–7. The prepared bigels were soft and smooth, with a color ranging from white to yellow based on the ratio of OL/WH.

**Figure 2:**
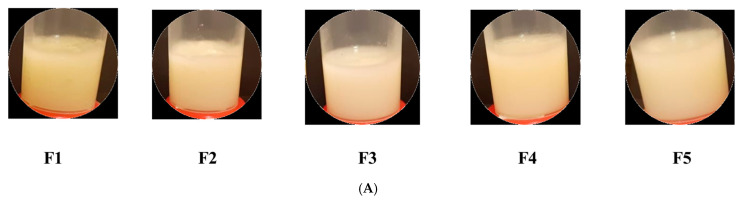

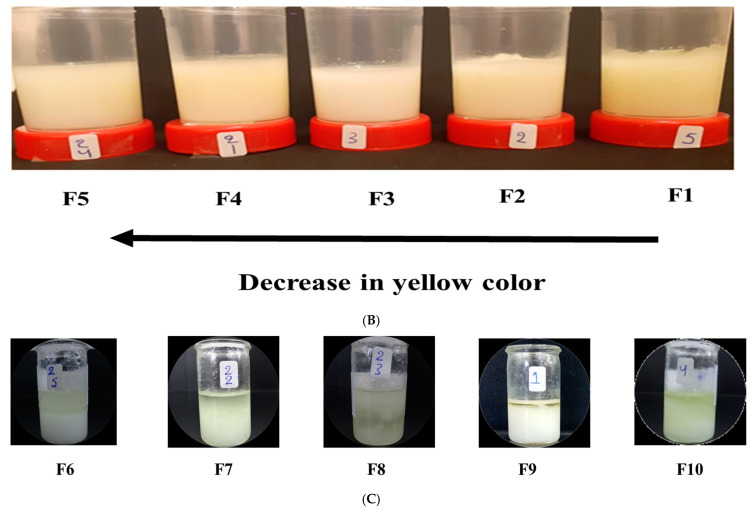
(**A**) Pictures of Type I bigels (F1–F5) showing no phase separation; (**B**) the range of yellow color of Type I bigels (F1–F5) which varied based on the proportion of OL; and (**C**) Type II bigels (F6–F10) showing phase separation and color change.

In this study, two types of bigels were prepared by mixing WH and OL at different ratios (1:1, 1:2, and 1:4). Type I bigels (F1–F5) were prepared by having the WH proportion either equal to or higher than OL. This type of bigel showed no phase separation or color change when tested visually (Figure 2A). The physical properties of the bigels were varied by altering the proportions of OL and WH, where the smoothness and consistency of the bigels increased with increasing the proportions of WH. At higher proportion of WH (F5 and F3, 1:4 OL:WH), bigels were easily spread and less sticky. On the other hand, the viscosity and firmness of the bigels increased with increasing the proportion of OL. Concerning the color of bigels, as the proportion of OL increased, the color changed from white to yellow (F1 > F2 = F4 > F3 = F5) (Figure 2B). In contrast, Type II bigels (F6–F10) were prepared by having an OL proportion either equal to or higher than WH. This type of bigel showed phase separation and color change when inspected visually (Figure 2C). Thus, they were excluded from further studies.

### 2.3. Characterization of Bigels

#### 2.3.1. Microstructural Characterization

**Figure 3:**
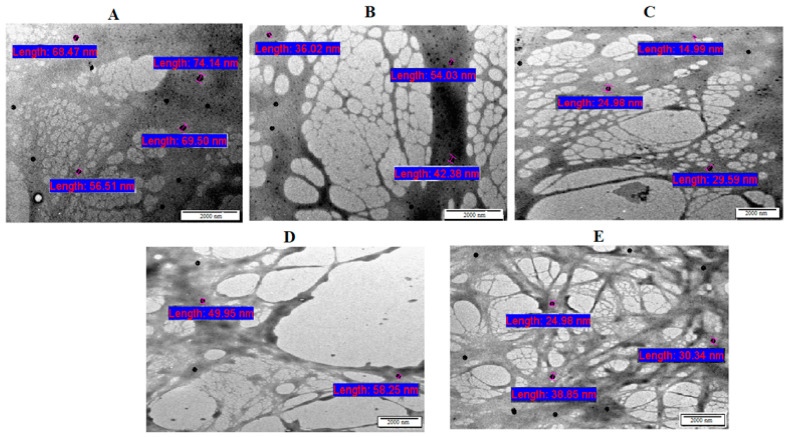
TEM images of bigels: (**A**) F1, (**B**) F2, (**C**) F3, (**D**) F4, and (**E**) F5.

The morphology of F1–F5 bigels was observed by TEM and a light-field microscope. TEM was used for imaging the network of the bigels and the distribution of CIP.HCl NE within the bigel network (Figure 3). F1 (OL:WH, 1:1) showed a structure formed from the dense EC fibers [28] that were enclosed with the honeycomb-like structure [29] formed from the less cross-linking, large pores of the CP network (Figure 3A). F2 (OL:WH, 1:2) has dense EC fibers of OL and a more visible honeycomb-like structure compared to F1 due to the higher proportion of WH (Figure 3B). F3 had the largest fraction of WH (OL:WH, 1:4), which increased the cross-linking density of the CP network and clearly displayed the honeycomb-like structure of the CP network, which was not covered by the EC fibers as in F1 (Figure 3C). F4 (OL:WH, 1:2) has less dense EC fibers and CP networks (Figure 3D). Although F2 and F4 have similar OL:WH ratios (1:2), their bigel networks varied based on the phase in which CIP.HCl NE was dispersed, whereas in F2, CIP.HCl NE was dispersed in OL, whereas in F4, CIP.HCl NE was dispersed in WH. F5, prepared from OL:WH (1:4), showed long, thin EC fibers and a honeycomb-like structure of small pores (Figure 3E). Although F3 and F5 have similar OL:WH ratios (1:4), a slight variation was observed in their networks, owing to the phase in which CIP.HCl NE was dispersed. Additionally, small spherical droplets of CIP.HCl NE with a size range of 14.99–74.14 nm were distributed within F1–F5 bigel networks (Figure 3). Although the size of CIP.HCl NE, detected by TEM, ranged between 22.29 and 32.07 nm, the increment in the size of CIP.HCl NE (14.99–74.14 nm) might be attributed to the increase in the viscosity of the medium after loading CIP.HCl NE into bigels. The same findings were demonstrated by Prasanth et al. [30] and Chakraborty et al. [31], who stated that adding thickening agents such as Carbopol, pectin, and sodium alginate resulted in an increase in the viscosity of the medium and hence a larger size of droplets. Nevertheless, the size of CIP.HCl NE loaded into bigels was between 10 and 100 nm, within the acceptable nanosized range of nanoemulsions [32].

**Figure 4:**
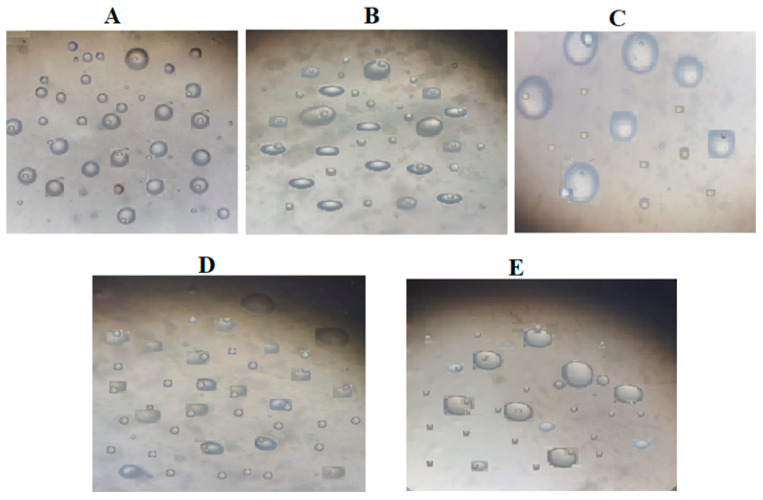
Light-field micrographs of: (**A**) F1, (**B**) F2, (**C**) F3, (**D**) F4, and (**E**) F5 observed under 40× magnification.

A light-field microscope was used to examine the homogeneity of bigels. Sudan-Black-B stain, used to stain fats and oils [33], was used to distinguish the OL phase from the WH phase. Figure 4 illustrates the light blue spheres of the OL phase dispersed into the continuous WH phase, conforming to Type I bigels (OL/WH). In addition, the smaller blue-dark spherical droplets of CIP.HCl NE were distributed within the bigel network, in agreement with that observed in the TEM images (Figure 3).

#### 2.3.2. Fourier Transform Infrared (FTIR)

The FTIR study was used to detect peaks in the chemical structure of materials. Any shifting, appearance, or disappearance of the characteristic peaks designates chemical interactions between bigel components.

**Figure 5:**
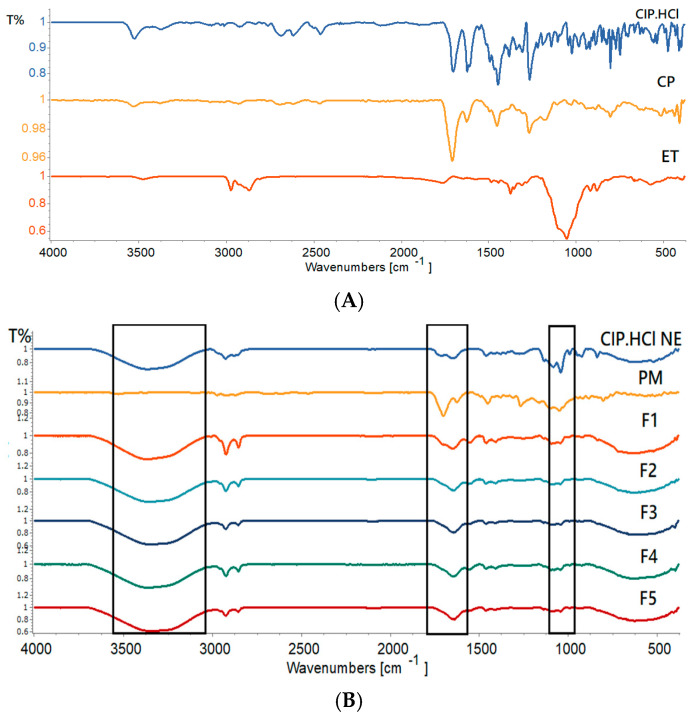
FTIR spectra of (**A**) CIP.HCl, CP, and ET, and (**B**) PM, CIP.HCl NE, and F1–F5 bigels.

Figure 5A illustrates the FTIR spectra of pure CIP.HCl, CP, and EC. The FTIR spectrum of CIP.HCl showed peaks of OH stretching vibration observed at 3525 cm^−1^, NH stretching vibration of the imino-moiety of the piperazine ring observed at 3372 cm^−1^, C=O stretching vibration observed at 1702 cm^−1^, and NH bending vibration of quinolone observed at 1621 cm^−1^. The OH bending vibration peak found at 1265 cm^−1^ confirmed the presence of carboxylic acid. Peaks in the 2924–3084 cm^−1^ region corresponded to alkene and aromatic C-H stretching, and those at 1445 cm^−1^ corresponded to the C-O stretching vibration. Moreover, the peak observed at 1023 cm^−1^ corresponded to the C-F group. These observations are in accordance with Sahoo et al. [34]. The FTIR spectrum of CP showed a well-defined peak for OH stretching vibration observed at 3529 cm^−1^, C=O stretching vibration observed at 1706 cm^−1^, and C-O-C of acrylates observed at 1268 cm^−1^. The peak in the 1419–1492 cm^−1^ region corresponded to the O-H/C-O bending vibration, and that at 1160 cm^−1^ corresponded to the ethereal crosslinking, representing a stretching vibration of the C-O-C group. The peak of C=CH was out-of-plane, bending between 850 and 800 cm^−1^. These observations are in accordance with Sahoo et al. [34]. The FTIR spectrum of EC showed a peak in the 2869–2973 cm^−1^ region, corresponding to the C-H stretching vibration. The OH stretching vibration peak was observed at 3476 cm^−1^, the C-O-C stretching vibration was observed at 1052 cm^−1^, and the C–H bending vibration was observed at 1374 cm^−1^. These observations are in accordance with the literature [24,35].

Figure 5B illustrates the FTIR spectra of PM, CIP.HCl NE, and F1–F5 bigels. The FTIR spectrum of PM, composed of CIP.HCl, EC, and CP, demonstrated the absence of any new peaks in the spectrum, indicating no chemical interaction between the drug and EC and CP. The functional groups of CIP.HCl were not clearly visible, as they might have been masked by the CP peaks. One peak was clearly distinct at 3529 cm^−1^, which corresponded to the OH stretching vibration peak, whereas the sharp peak observed at 1703 cm^−1^ corresponded to the C=O stretching vibration peak. The peak observed at 1449 cm^−1^ corresponded to the OH/CO bending vibration peak, and that at 1266 cm^−1^ corresponded to the C-O-C of acrylate. The functional groups of EC clearly existed, where the peak in the 2869–2972 cm^−1^ region corresponded to the CH stretching vibration and that at 1049 cm^−1^ corresponded to the C-O-C stretching vibration.

The FTIR spectrum of CIP.HCl NE demonstrated the CH stretching vibration peak at 2857–2924 cm^−1^. The C=O peak was shifted from 1702 to 1645 cm^−1^, suggesting hydrogen bonding between the oxygen atom of the drug and the aqueous phase of NE [36]. Moreover, the broad peak observed at 3362 cm^−1^ corresponded to the OH bond stretching of the continuous phase of CIP.HCl NE.

The FTIR spectra of bigels varied based on the ratio of OL:WH in F1–F5. The characteristic peaks of CIP.HCl, CP, and EC were more noticeable in the spectrum of PM than in the bigels spectra. The C=O stretching vibration peaks of F1–F5 bigels shifted to 1652, 1645, 1642, 1642, and 1636 cm^−1^, respectively, indicating the formation of hydrogen bonding [36]. Additionally, the broad OH peak in F1–F5 bigels shifted to 3367, 3351, 3348, 3360, and 3344 cm from 3344 cm^−1^, respectively, which corresponded to the OH groups and intermolecular hydrogen bonding [34]. It was found that the OH stretching peaks in bigels with a higher WH proportion were shifted to a lower frequency. Peaks observed between 2853 and 2924 cm^−1^, corresponded to the C-H stretching vibration of EC in the OL phase, which became more distinct as the ratio of OL was increased in bigels. In addition, the C-O-C stretching peak at 1049 cm^−1^, observed in EC, was shifted to 1042–1043 cm^−1^ in bigels. The downward shift in these peaks is due to reducing the bond strength and force constant of the C-O-C bond of EC in OL [35]. Table 3 summarizes the characteristic peaks in the FTIR spectra of F1–F5 bigels.

#### 2.3.3. Rheological Studies

##### Viscosity of Bigels

**Figure 6:**
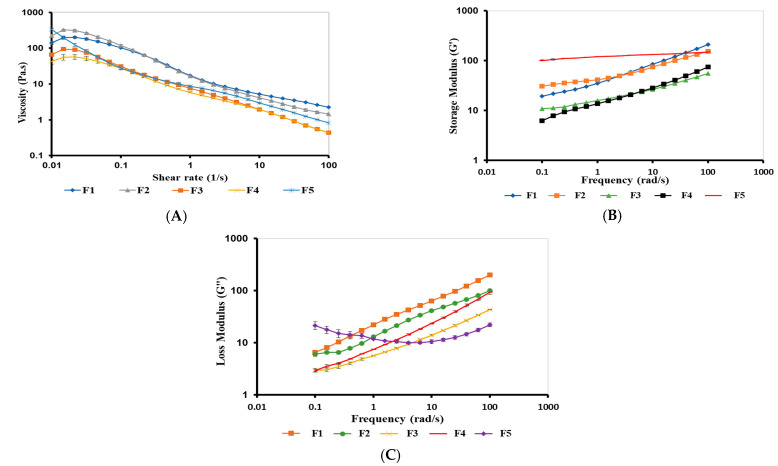
(**A**) Viscosity curves, (**B**) elastic G′ modulus of F1, F2, F3, F4, and F5 bigels, and (**B**) elastic G′ and (**C**) viscous G″ moduli of F1–F5 bigels. The data are presented as mean ± SD (*n* = 3).

The viscosity of topical formulations influences skin feeling, spreadability of formulation, and penetration of drugs into the skin [37]. F1–F5 bigels showed a pseudoplastic (shear-thinning) flow, where the viscosity decreased with shear rate (Figure 6A), in accordance with Hamed et al. [9,38]. The viscosity of bigels varied based on the OL:WH ratio and the dispersion of CIP.HCl NE in either the WH or OL phases. The viscosity of bigels increased as the proportion of OL increased. This is in accordance with Singh et al. [39], who reported that bigels with higher OL content exhibited higher viscosity. Thus, the viscosities of F1 and F2 were higher than those of F3, owing to the high OL proportion (1:1 and 1:2 vs. 1:4, OL:WH). In comparison to F1–F3, F4 exhibited the lowest viscosity. Although the OL proportion in F4 was high (1:2, OL:WH), its lowest viscosity might be attributed to the dispersion of CIP.HCl NE in the WH phase. Similarly, F5 exhibited a higher viscosity than F3, suggesting the formation of a highly connected network between CIP.HCl NE and WH phases, which enhanced the viscosity of the bigel network. The pseudoplastic behavior of bigels implied that when bigels are subjected to shear forces during application, they become less viscous, enabling easier spreading of the bigel on the skin’s surface, better contact with the skin, and a more uniform application that ultimately improves permeation properties [40,41].

##### Strain-Sweep of Bigels

The strain-sweep test is conducted to determine the LVR of bigels, where G′ and G″ are constant within this region, indicating that the bigel structure would remain undamaged during the frequency-sweep tests [7]. The maximum strain of LVR is called a critical strain (γ_C_). After γ_C,_ a reduction in G′ and G″ is observed, indicating a termination of the LVR and a rupture in the bigel network [9]. The LVR, applied strain within the LVR, and γ_C_ of F1–F5 are summarized in Table 4.

##### Frequency Sweep of Bigels

Bigels exhibited viscoelastic properties, with G′ dominating G″ over 0.1–100 rad/s (Figure 6B, C). G′ is the elastic (solid) component of the bigels, whereas G″ is the viscous (liquid) component of the bigels [38]. It was found that G′ and G″ of bigels varied based on the ratio of OL: WH, in agreement with the viscosity data. The viscoelastic properties of F1–F3 increased with an increase in the OL ratio, where F1 and F2 showed higher G′ and G″ compared to those of F3 (1:1, 1:2 vs. 1:4 OL:WH). For F4 and F5 (1:2 and 1:4, OL:WH), where the CIP.HCl NE was dispersed in the WH phase, the viscoelastic properties varied based on the ratio of WH. Therefore, increasing the WH ratio in the bigel resulted in an increase in G′ and G″. Hence, the viscoelastic properties of F5 were higher than those of F4. This might be attributed to the formation of a strong CP network upon the addition of the CIP.HCl NE to the WH phase. The viscoelastic properties of bigels with elastic modulus dominating viscous modulus (G′ > G″), implied that bigels tend to recover their stiffness after shear stress is eliminated, enabling them to adhere to the skin for a longer period of time and control drug release [42,43]. Moreover, the bigel matrix can act as a physical barrier, protecting the drug from environmental variables such as temperature changes and hence contributing to long-term stability [44].

#### 2.3.4. In Vitro Release Studies

**Figure 7:**
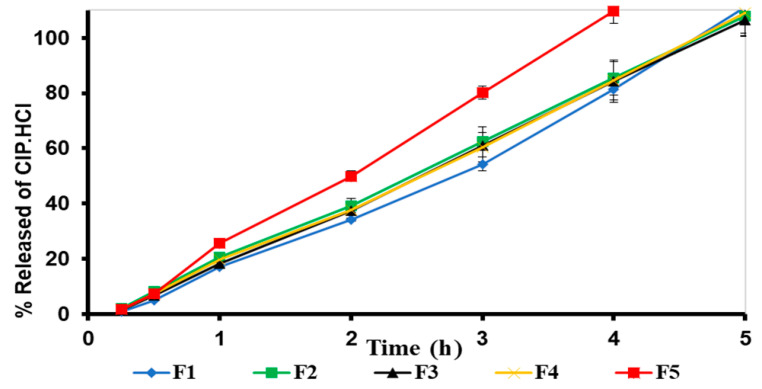
In vitro release profiles of F1–F5 bigels through the cellulose membrane. The data are presented as the mean ± SD (*n* = 3).

To guarantee sink conditions, 5% acetic acid was selected as a receiving medium due owing the high solubility of CIP.HCl in 5% acetic acid. The cumulative amount of drug release (%Q) from F1 to F5 bigels was plotted versus time (Figure 7). The CIP.HCl release from F1 to F5 was controlled, as was the rate of CIP. HCl release varied based on the ratio of OL:WH. For F1–F4, the drug release profiles were superimposed on the cumulative amount of CIP.HCl increased with time, attaining complete release after 5 h. Whereas F5 (OL:WH, 1:4) showed a faster release rate compared to those of F1–F4, where complete drug release was attained after 4 h. The fast release in F5 might be attributed to the high proportion of WH and the dispersion of NE in the WH phase, which enhanced the drug release rate. Whereas in F3 (OL:WH, 1:4), where the CIP.HCl NE was dispersed in the OL phase, the drug was trapped in OL, retarding the CIP.HCl release from the bigel network for an extra one hour.

The cumulative amount of CIP.HCl released per unit surface area of cellulose membrane after 5 h (Q5) was 568.1 ± 33.5, 589.6 ± 51.9, 576.3 ± 48.7, and 602.4 ± 54.9 µg/cm^2^ for F1–F4, respectively. For F5, the accumulative amount of CIP.HCl permeated after 4 h (Q4) was 603.2 ± 35.0 µg/cm^2^. The flux (J) of F1–F5 bigels was 115.3, 120.7, 119.7, 123.0, and 157.8 µg/cm^2^/h, respectively.

#### 2.3.5. Mechanism of CIP.HCl Release

The mechanism of CIP.HCl release from F1 to F5 bigels was determined using the Korsmeyer–Peppas kinetic model as described [45]. The mechanism of release (n), coefficient of determination (R^2^), and release rate constant (K, h^−1^) are summarized in Table 5. The Korsmeyer–Peppas model was fitted with the CIP.HCl release from bigels with R^2^ ranged between 0.9895 and 0.9988. The diffusion mechanism (n) of F1–F4 was ~1, indicating a zero-order release mechanism where the release was constant with time and independent of the CIP.HCl concentration. Whereas for F5, n was 1.12, indicating a super-case II transport that involves polymer relaxation and diffusion [9].

#### 2.3.6. Antimicrobial Study

##### Well Diffusion Methods

The antimicrobial activity of CIP.HCl NE and F1–F5 bigels was evaluated by measuring the inhibitory effects against Gram-negative (*E. coli*, *P. aeruginosa*, and *P. mirabilis*) and Gram-positive (*B. subtilis* and *S. aureus*) bacteria (Figure S3). The diameters of the zones of inhibition are shown in Table 6. Wells 1 and 2 were filled with the positive controls (0.3% Ciprocin eye/ear drops and 1.5% CIP.HCl solution in DW). Wells (3, 4, 5, 6, 7, and 8) were filled with CIP.HCl NE and F1–F5, respectively. Wells (3N, 4N, 5N, 6N, 7N, and 8N) were filled with blank samples of NE and F1–F5, respectively. The zones of inhibition were visible for CIP.HCl NE, 1.5% CIP.HCl solution, and bigels. This suggests that CIP.HCl NE has effective antibacterial activity when incorporated into the bigels. Interestingly, the blank nanoemulsion showed an antimicrobial zone of inhibition due to the presence of 5% acetic acid in the formulation. It has been reported that acetic acid might exhibit antimicrobial activity at low concentrations (0.5%), owing to the dissociation of acetic acid within the microbial cells, which causes metabolic disruption and a drop in the intracellular pH [46].

Additionally, F1–F5 showed antimicrobial activity against both Gram-negative and Gram-positive bacteria. Comparing the antimicrobial activity of the positive control with that of bigels, it was found that the antimicrobial activity of 0.3% Ciprocin was nearly equal to that of the bigels. Moreover, 0.3% Ciprocin eye/ear drops, 1.5% CIP.HCl solution, 1.5% CIP.HCl NE, and F1–F5 showed statistically higher zones of inhibition compared to blank NE against all bacterial strains, as shown in Table 6 (* *p* < 0.05, ** *p* < 0.01, *** *p* < 0.001). Finally, blank F1–F5 bigels showed no antimicrobial activity with zones of inhibition of <10 mm.

##### Ex Vivo Skin Infection Model

The antimicrobial activity of F3 and F5 was evaluated using the skin infection model where skin pieces removed from male mice, were infected with Gram-negative (*E. coli*, *P. aeruginosa*, and *P. mirabilis*) and Gram-positive (*B. subtilis* and *S. aureus*) bacteria. Treatments of F3 and F5 bigels were applied to skin pieces, and the number of colony-forming units (CFU) per skin piece was measured after incubation and compared to the positive control. Additionally, pieces of skin were used to ensure no contamination or growth of other bacteria from the external environment as a negative control (Table 7). F3 and F5 showed antimicrobial activity against Gram-negative and Gram-positive bacteria (Figure S4). After 24 h, the log10 reduction for F3 was between −7.04 and −9.55 and between −5.35 and −9.58 when compared to the positive control against Gram-negative and Gram-positive bacteria, respectively. Whereas the log10 reduction for F5 was between −9.63 and −9.74 and −9.58 and −9.76 when compared to the positive control against Gram-negative and Gram-positive bacteria, respectively. These results demonstrated that F3 and F5 bigels exhibited higher antimicrobial activity than the positive control against all bacterial strains (Table 7, *p* < 0.001). Moreover, F5 bigel showed a higher inhibitory effect against *B. subtilis* compared to F3 (*p* < 0.0001). Thus, this suggests a good correlation between the results of the skin infection model and those of well-diffusion methods.

#### 2.3.7. Rheological Stability of Bigels

The rheological stability of F1–F5 bigels was investigated after their storage for 3 months at room temperature and compared to those reported initially.

##### Viscosity of Bigels

**Figure 8:**
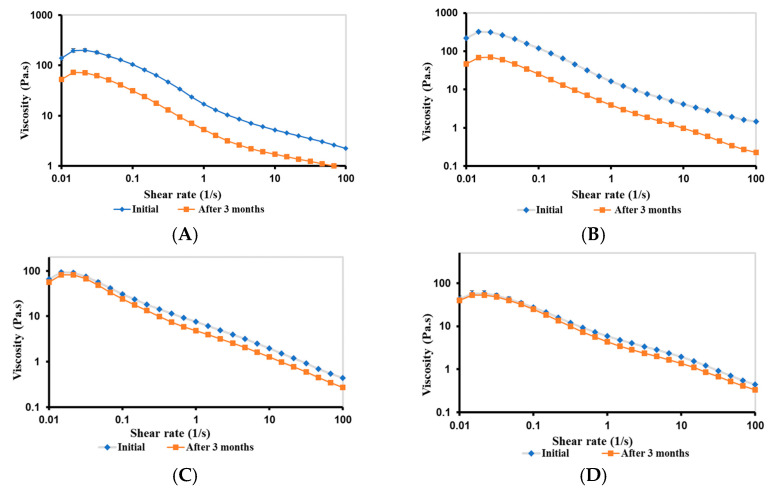

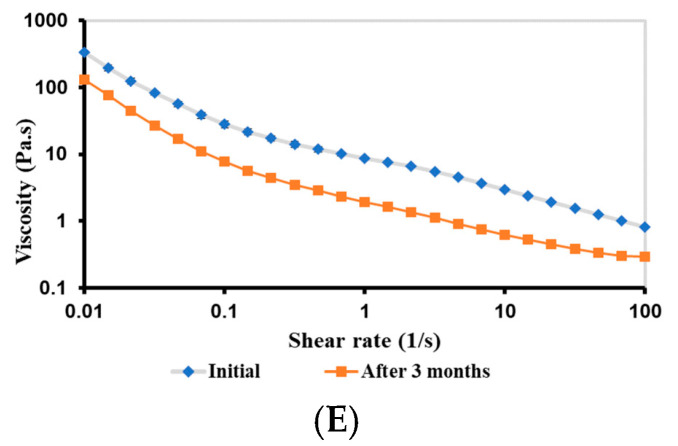
Viscosity curves of (**A**) F1, (**B**) F2, (**C**) F3, (**D**) F4, and (**E**) F5 bigels after three months of storage at room temperature. The data are presented as mean ± SD (*n* = 3).

Figure 8 shows a comparison of the viscosity curves of F1–F5 bigels reported initially and those reported after three months. The viscosity of F1 (Figure 8A) and F2 (Figure 8B) dramatically decreased over time, indicating a less stable network after three months. This can be explained by the agglomeration of the small droplets of CIP.HCl NE and the large droplets of oil in OL, forming a weak physical network that initially enhanced the viscosity of bigels. However, after three months, this network becomes weaker and less stable, leading to a decrease in the viscosity of bigels [47,48]. Contrarily, the viscosity of F3 (Figure 8C) and F4 (Figure 8D) slightly reduced after three months, indicating a more stable continuous CP network. Finally, the viscosity of F5 decreased after three months, suggesting a weaker bigel network (Figure 8E).

##### Viscoelastic Properties of Bigels

**Figure 9:**
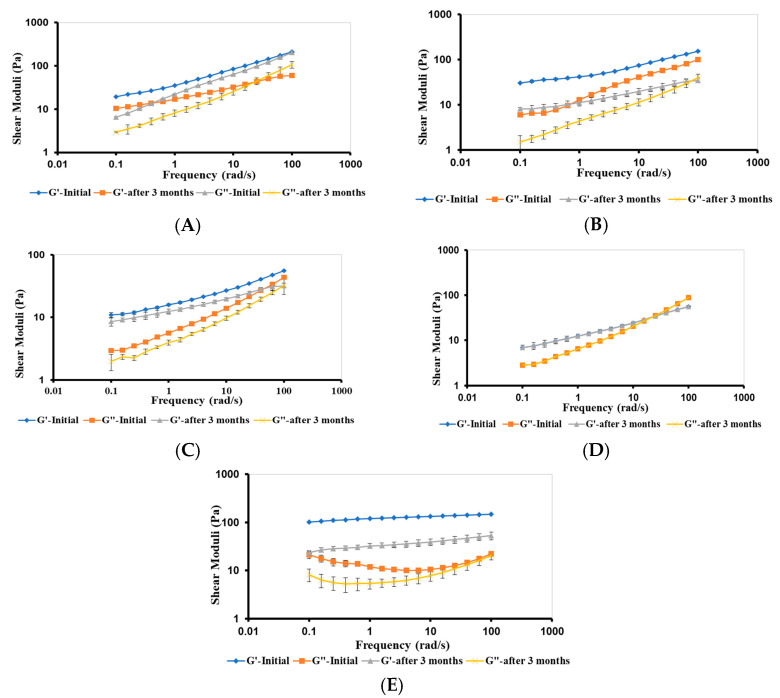
Frequency-dependence (G′ and G″) of (**A**) F1, (**B**) F2, (**C**) F3, (**D**) F4, and (**E**) F5 bigels after three months of storage at room temperature. The data are presented as mean ± SD (*n* = 3).

After three months of storage, G′ and G″ of bigels decreased, except for F4, which retained its viscoelastic properties, suggesting a stable CP network (Figure 9). The degree of reduction in G′ and G″ varied based on the composition of the bigels. These results corroborated with the viscosity results of the bigels (Figure 8).

#### 2.3.8. Physicochemical Stability

Bigels are generally subjected to stability studies as biphasic systems are prone to instability over time [10]. For thermodynamic stability, F1–F5 bigels were subjected to five freeze/thaw (−20/70 °C) cycles. F1 showed a phase separation after two months; hence, it was excluded from stability studies. Whereas F2–F5 retained their structural integrity, showing no phase separation and indicating that these bigels were thermodynamically stable.

The physical and chemical stability of F2–F5 bigels was tested at various storage conditions for three months. F2–F5 maintained their color range, ranging from white to a slight yellow color. Additionally, bigels showed a reduction in pH over time, ranging from 5.7 to 6.5 (Table 8). Moreover, stability studies showed that F2 and F4 were less stable, where phase separation was observed after three months at 2–8 °C, room temperature, 30 °C/65% RH, and 40 °C/75% RH. Whereas F3 and F5 remained stable over three months under various storage conditions. The CIP.HCl content in bigels at various storage conditions after three months is summarized in Table 9. The data showed that F5 had the highest drug content over time, whereas F2–F4 showed a remarkable reduction in drug content. These observations warrant further investigation.

## 3. Conclusions

The CIP.HCl NE-loaded Type I bigels (F1–F5, OL-in-WH), prepared at various OL to WH ratios (1:1, 1:2, and 1:4), were successfully developed. The CIP.HCl NE was dispersed in either the OL or WH phases. The microscopic analysis revealed a variation in the microstructure of bigels based on the OL to WH ratio. Bigels displayed pseudoplastic behavior and viscoelastic properties, with an elastic modulus dominating the loss modulus. The release of CIP.HCl from bigels provided a controlled-release pattern followed by a zero-order kinetic release for F1–F4 and a supercase II transport mechanism for F5. Additionally, the antimicrobial activity of the bigels was confirmed when compared with the commercially available CIP.HCl eye/ear drops and CIP.HCl solution. The F5 bigel was the most stable among other bigels, maintaining its appearance and color under various storage conditions for three months. This study showed that the comparative evaluation of bigels varied based on the ratio of OL to WH, suggesting that the physicochemical, microstructural, rheological, and antimicrobial properties, in vitro release, and stability of bigels could be tailored by altering the OL:WH ratio. In summary, the two drug delivery systems of O/W nanoemulsion and bigel could potentially be used for the topical delivery of antimicrobial drugs for skin infections. This is because the drug loaded nanoemulsion retained its antimicrobial activity and released it at a controlled rate after incorporation into bigels. Together, CIP.HCl NE-loaded Type I bigels (OL-in-WH) could improve the clinical outcomes of CIP.HCl in treating skin infections by enhancing drug penetration into skin and drug antimicrobial activity, as demonstrated by the in vitro well diffusion method and the ex vivo skin infection model. Nevertheless, there remain certain limitations, such as the physical instability of Type II bigels (WH-in-OL) and the crystallization of CIP.HCl NE when stored in the refrigerator, that warrant further studies.

## 4. Materials and Methods

### 4.1. Materials

Ciprofloxacin HCl (CIP.HCl, Zhejiang Huayi Pharmaceutical Co., Jinhua, China) was kindly provided by Hikma Pharmaceuticals (Amman, Jordan). Ciprocin Eye/Ear Drops (0.3%, Amman Pharmaceuticals CO., Amman, Jordan) were purchased from the market. Ethocel (Ethyl cellulose, EC) was a gift from Colorcon Limited (Dartford, UK), and Acrypol*^®^*971P (CP) was obtained from Corel Pharma Chem (Ahmedabad, India). Oils (oleic acid (OA) was obtained from Fisher Scientific UK (Loughborough, UK) and isopropyl myristate (IPM) from Evonik (Essen, Germany)). Cosurfactants (propylene glycol (PG) was obtained from Dow Chemicals (Midland, MI, USA), ethanol 96% from GPR Rectapur VWR (Darmstadt, Germany), and polyethylene glycol 400 (PEG 400) from INEOS (Rolle, Switzerland)). Surfactants (Tween^®^20 (TW-20) was obtained from Tedia (Fairfield, OH, USA), and Labrasol^®^ was generously provided by Gattefosse (Saint Priest Cedex, France)). Triethanolamine (TEA) was obtained from INEOS (Rolle, Switzerland), acetic acid from Acros Organics-Fisher Scientific (Pittsburg, PA, USA), and acetonitrile (HPLC grade) from CARLO ERBA Reagents (Val-de-Reuil, France). Nutrient agar was obtained from NA, Biolab (Budapest, Hungary), and Mueller-Hinton agar medium from MHA, Oxoid (Wade Road, UK). Sudan-Black-B was provided by Sigma-Aldrich Chemical Co. (St. Louis, MO, USA). Other chemicals were utilized as they were received.

### 4.2. Methods

#### 4.2.1. Preparation of Ciprofloxacin HCl Nanoemulsion (CIP.HCl NE)

##### Solubility of CIP.HCl

The solubility of CIP.HCl was determined in nanoemulsion components as follows: OA and IPM as oil phases; TW-20 and Labrasol^®^ as surfactants; PG, PEG 400, and ethanol as cosurfactants; and 5% acetic acid and water as aqueous phases, as described in [49]. Briefly, an excess amount of CIP.HCl was dissolved in 3 mL of tested samples placed in 10 mL screwed-on test tubes. Samples were mixed using a vortex mixer and placed in a water bath at 37 °C for 24 h. Then samples were centrifuged at 3500× *g* for 15 min (Hermle Z326K centrifuge, Wehingen, Germany), and the supernatants were appropriately diluted with 0.1 N HCl. The concentration of CIP.HCl was assessed by UV-Vis spectrophotometry (Varian, Cary UV/Vis spectrophotometer) at a wavelength (λ_max_) of 277 nm, using a standard calibration curve of CIP.HCl prepared in 0.1 N HCl.

##### Preparation of CIP.HCl NE

An O/W CIP.HCl NE (1.5%) was prepared by spontaneous emulsification as described [7]. Briefly, to prepare 3 mL of 1.5% CIP.HCl NE, an oil (6.67% *v*/*v*, 0.2 mL) and a surfactant/cosurfactant mixture (60.0% *v*/*v*, 1.8 mL) were mixed for <1 min to obtain a clear mixture. Then, 45 mg CIP.HCl was dissolved in 5% acetic acid (33.3% *v*/*v*, 1 mL) for about 3 min and then titrated into the oil/(surfactant/cosurfactant) mixture with continuous mixing with a vortex for <1 min. Finally, CIP.HCl NE was kept in the sonicator at room temperature for 30 min to achieve a clear nanoemulsion.

#### 4.2.2. Characterization of CIP.HCl NE

##### Particle size, Polydispersity Index, and Zeta Potential

The droplet size, polydispersity index (PDI), and zeta potential (ζ-potential) of CIP.HCl NE were measured using a nanosizer (Nicomp Nano Z3000, Santa Barbara, CA, USA). A total of 0.5 mL of CIP.HCl NE was diluted in 4.5 mL of distilled water (DW). Three measurements were obtained.

##### Morphology of CIP.HCl NE

Transmission electron microscopy (TEM, FEI Morgani 268, Eindhoven, Netherlands) connected to a digital camera (Olympus Mega View II, Olympus Soft Imaging Solutions GmbH, Münster, Germany) was used to determine the size and morphology of CIP.HCl NE. Briefly, one drop of CIP.HCl NE was diluted with six drops of DW and gently mixed. Before observing under TEM, a one-drop sample was placed on a copper grid coated with carbon and left to dry.

#### 4.2.3. High Performance Liquid Chromatography

The amount of CIP.HCl was determined using a high performance liquid chromatography (HPLC) as described [50]. The HPLC (Thermo Scientific™, Dionex UltiMate™ 3000, Potsdam, Germany) was fitted with a UV/Vis detector. The chromatographic separation was performed using an RP-C18 column (250 mm × 4.6 mm, 5 µm). A mobile phase composed of 0.025 M phosphoric acid (adjusted by TEA to pH 2.6) and acetonitrile (80:20 *v*/*v*) was delivered at a flow rate of 1.5 mL/min, and the sample injection volume was set at 20 μL. The detection wavelength was 278 nm, and the retention time of the CIP.HCl peak was detected at 6.5 min. A standard calibration curve of CIP.HCl, prepared in the mobile phase with a linear equation of y = 113,653x + 6528.3 (R^2^ = 0.9997) and built at a concentration range of 5–20 µg/mL, was used to determine the concentration of CIP.HCl in tested samples.

#### 4.2.4. Determination of CIP.HCl in Nanoemulsion

To determine CIP.HCl content in NE, 0.5 g of CIP.HCl NE, equivalent to 7.5 mg of CIP.HCl, was placed in 50 mL volumetric flasks. A quantity of 30 mL of 5% acetic acid was added to CIP.HCl NE. The flasks were placed on a magnetic stirrer plate and mixed for 20 min, then 10 mL of DW were added to the flasks and stirred for another 15 min. The volume was continued up to 50 mL with DW, and the flasks were placed in a sonicator at 37 °C for 15 min, to ensure complete dissolution. The samples were then filtered using a 0.45 µm syringe filter, and 0.5 mL of filtered samples were diluted by 9.5 mL of mobile phase and injected into the HPLC system at 278 nm. The assay of CIP.HCl NE was repeated three times.

#### 4.2.5. Stability Studies of CIP.HCl NE

##### Thermodynamic Stability Studies

The CIP.HCl NE was subjected to centrifugation, heating/cooling cycles, and freezing/thawing cycles as described in [9]. Initially, CIP.HCl NE was centrifuged at 3500× *g* for 15 min. If CIP.HCl NE showed no phase separation, it was subjected to six heating/cooling (45/4 °C) cycles with storage at each temperature of not less than 48 h. If the CIP.HCl NE remained clear, then it was frozen (−21 °C) and thawed at room temperature three times, with storage at each temperature of not less than 48 h. Each experiment was repeated three times.

##### Chemical Stability

Stability studies were determined by storing CIP.HCl NE in the refrigerator (2–8 °C), at room temperature (22 °C), 30 °C/65% relative humidity (RH), and 40 °C/75% RH. Samples were withdrawn at regular intervals of 1, 2, and 3 months and evaluated for CIP.HCl content using the HPLC method. The CIP.HCl content was determined initially and compared to that of the stored samples.

#### 4.2.6. Preparation of Bigels

##### Preparation of Bare WH

A weighed amount of Carbopol (CP) was added to a specific volume of DW and stirred for 1 h at room temperature, then kept overnight until a CP dispersion was obtained. Then, the pH was adjusted to 9–10 using a few drops of TEA and measured using a pH meter (Jenway, model 3510, Staffordshire, UK).

##### Preparation of Bare OL

A weighed amount of ethyl cellulose (EC) was added to a specific volume of OA maintained at 140–150 °C and stirred at 500 rpm until a clear mixture was obtained.

##### Preparation of Bigels

Type I (OL/WH) bigels (F1–F5) were prepared by mixing OL and WH at different ratios (1:1, 1:2, 1:4, OL:WH). For F1–F3 bigels, the CIP.HCl NE was added gradually into the OL phase and dispersed for <1 min, whereas for F4–F5 bigels, the CIP.HCl NE was added gradually and dispersed into the WH phase and dispersed for <1 min. Type II (WH/OL) bigels (F6–F10) were prepared by mixing different ratios of WH and OL (1:1, 1:2, and 1:4, WH/OL). For F6–F8 bigels, the CIP.HCl NE was added gradually into the WH phase and dispersed for <1 min, whereas for F9–F10 bigels, the CIP.HCl NE was added gradually into the OL phase and dispersed for <1 min. In detail, F1–F3 and F9–F10 bigels were prepared by dispersing 20 mL of CIP.HCl NE into OL, maintained at 140–150 °C. The dispersion was then added gradually and at a high shear rate (1000 rpm) to the bare WH. The pH of bigels was adjusted to 6–7 with a few drops of TEA. Whereas F4–F5 or F6–F8 were prepared by dispersing 20 mL of CIP.HCl NE into WH at room temperature. The dispersion was then added gradually and at a high shear rate (1000 rpm) to the bare OL, maintained at 140–150 °C. The pH of the bigels was adjusted to 6–7 with a few drops of TEA. Table 10 and Table 11 illustrate the composition of Type I and Type II bigels, respectively, and Figures S5 and S6 represent the schematic diagram of the preparation of Type I and Type II bigels, respectively.

#### 4.2.7. Characterization of CIP.HCl Bigels

##### Physical Appearance

Bigels were evaluated for physical stability, including phase separation, grittiness, and breakdown (cracking). Bigels that showed physical instability were excluded from further studies.

##### Microstructural Characterization

Light-field microscopy (Optika, model X-LED with a white LED microscope, Ponteranica, Italy) was used to study the microstructure of bigels and differentiate between the types of bigels using Sudan-Black-B stain. Additionally, TEM (FEI Morgani 268, Eindhoven, The Netherlands) was used to study the microstructure of bigels. Briefly, one drop of each bigel was diluted with twenty drops of DW and gently mixed. Before observing under the TEM, a one-drop diluted sample was placed on a copper grid coated with carbon and left to dry.

##### Fourier Transform Infrared (FTIR)

The FTIR spectra were performed for CIP.HCl powder, EC, CP, bigels F1–F5, and physical mixture (PM) of bigels with ratios close to those of the prepared bigels (OL: WH, 1:4). The FTIR (Vertex 70 spectrometer, Bruker, Germany) measurements were carried out over a frequency range between 3500 and 500 cm^−1^ with 10 scans and 4 cm^−1^ resolutions. The analysis of the spectrum data was performed using Spectragryph (Version 1.2.16.1).

#### 4.2.8. Determination of CIP.HCl in Bigels

The CIP.HCl content in bigels was determined using the HPLC method. Briefly, a bigel sample of 0.5 g, equivalent to 1.5 mg CIP.HCl, was placed in 50 mL volumetric flasks and placed on a hotplate stirrer. A 10 mL DW was added to the flasks and stirred for 15 min. Using DW, the volume was continued to 50 mL. To ensure proper agitation, flasks were placed in a sonicator at 37 °C for 15 min, and the samples were filtered using a 0.45 µm syringe filter. Finally, 0.5 mL of filtered sample was diluted by 1.5 mL of mobile phase and injected into the HPLC system at 278 nm for analysis. The assay of bigels was repeated three times.

#### 4.2.9. Rheological Studies

##### Viscosity Curves

The viscosity of bigels was determined at 32 °C, to mimic skin surface temperature [37], using a controlled-stress rheometer (CSR) (MCR 302, Anton Paar, Graz, Austria) with a cone/plate geometry of 50 mm and a 1.010° cone angle, as described [51]. Prior to analysis, a 0.5 g bigel was loaded on the plate and allowed to relax for 1 min. The viscosity of bigels was measured at shear rates of 0.1–100 s^−1^. At least three independent samples were used in the viscosity studies.

##### Strain-Sweep

Strain-sweep studies of bigels were conducted to determine the linear region of viscoelastic properties (LVR) as described in [51]. The elastic modulus (G′) and viscous modulus (G″) of bigels were probed at a strain range of 0.01–100%. The CSR with 50 mm cone/plate geometry and a 1.010° cone angle was used in these experiments. The temperature of the cone/plate was kept at 32 °C using a Peltier temperature control system. A 0.5 g bigel sample was loaded on the plate, left to relax for 1 min, and equilibrated at 32 °C. Then the cone oscillated at 6.28 rad/s. At least three independent samples were used in the strain-sweep studies.

##### Frequency-Sweep

The frequency-sweep studies of bigels were carried out at a strain value selected within the LVR and a frequency of 0.1–100 rad/s, as described in [51]. The CSR with 50 mm cone/plate geometry and a 1.010° cone angle was used to conduct the frequency-sweep studies at 32 °C. At least three independent samples were used in the frequency-sweep studies.

#### 4.2.10. In Vitro Release

The in vitro release studies were carried out using Franz diffusion cells (Copley Scientific Ltd., Nottingham, UK) at 32 °C with a 1.76 cm^2^ surface area and a 6 mL receiving compartment. Cellulose membrane (MWCO 12,000–14,000 Da) was soaked in 5% acetic acid overnight before use. The membrane was placed between the donor and receiving compartments of the diffusion cells. The receiving compartment was filled with 5% acetic acid to solubilize CIP.HCl and ensure sinking conditions. During the experiment, the contents of the receiving compartments were stirred at 500 rpm. A bigel sample of 0.3 was placed in the donor compartment. After 0.25, 0.5, 1, 2, 3, 4, 5, and 6 h, volumes of 1 mL solutions in the receiving chambers were withdrawn. A 1 mL volume, equal to that withdrawn, of fresh medium thermostated at 32 °C was added to the receiving compartment to ensure sink condition. The CIP.HCl concentration was determined by UV-Vis spectrophotometry at λ_max_ 277 nm. Three measurements were performed for each bigel. The cumulative amount of CIP.HCl released (%Q) was plotted versus time. The CIP.HCl flux was calculated from the slope of the linear portion of the cumulative amount permeated through the membrane per unit area (µg/cm^2^) versus time.

#### 4.2.11. Kinetic Release Analysis

The mechanism of CIP.HCl release from bigels was assessed by fitting the in vitro release data into the Korsmeyer–Peppas mathematical model [45]:

Q = Kt^n^

where Q is the amount of CIP.HCl release obtained from the ratio of the cumulative amount of CIP.HCl released at time t and that released at infinite time, K is the release rate constant, and n is the diffusion exponent, where n = 0.5 designates Fickian diffusion, 0.5 < n < 1.0 designates non-Fickian transport, n = 1.0 designates zero-order release, and n > 1 designates supercase II transport (polymer relaxation and diffusion) [45]. The fractional CIP.HCl release (Q) was calculated between 15 and 60% [52].

#### 4.2.12. Antimicrobial Studies

##### Microorganisms

The Gram-positive bacteria (*Bacillus spizizenii* (*B. spizizenii*) ATCC 6633, *Staphylococcus aureus* (*S. aureus*) ATCC 6538), and Gram-negative bacteria (*Escherichia coli* (*E. coli*) ATCC 8739, *Pseudomonas aeruginosa* (*P. aeruginosa*) ATCC 12453, and *Proteus mirabilis* (*P. mirabilis*) ATCC 43071) used in this study were obtained from the American Type Culture Collection (ATCC). The bacterial strains were grown at 37 °C and maintained on nutrient agar.

##### Well-Diffusion Method

The antimicrobial activity of bigels was tested in vitro against Gram-positive and Gram-negative bacteria by the Kirby–Bauer method [53]. Mueller-Hinton agar was used as a medium. Bacterial inoculum was prepared in 5 mL phosphate-buffered saline (PBS) (0.5 McFarland standards), where 100 µL of bacterial suspension was inoculated on fresh Mueller-Hinton agar using a cotton swab. Wells were then bored on the Muller-Hinton agar plates using a sterilized borer. Each well was filled with 30 µL of 0.3% bigels and 1.5% CIP.HCl NE, positive controls (1.5% CIP.HCl solution and 0.3% Ciprocin eye/ear drops), a negative control of DW, and blank samples of bigels and nanoemulsion. The inoculated plates with various pathogenic bacteria were incubated at 37 °C for 18 to 24 h. The test was repeated twice for each sample.

##### Ex Vivo Skin Infection Model

The antimicrobial activity of bigels (F3 and F5) was tested in vitro against Gram-positive and Gram-negative bacteria using the ex vivo skin infection model [54]. Five colonies of each tested bacteria were taken from the nutrient agar plates, inoculated into 5 mL of Muller-Hinton broth, and incubated overnight at 37 °C. The bacteria were diluted in 5 mL Mueller-Hinton broth, and the cell density was adjusted to 1.5 × 10^8^ CFU/mL (0.5 MacFarland standard). Then, the bacterial suspension was diluted with normal saline to obtain a final inoculum of 10^3^–10^7^ CFU/mL. This inoculum was used immediately after preparation to infect the skin pieces.

In this experimental work, male rat skin was used. The Research Ethics Committee at Al-Zaytoonah University of Jordan has reviewed and approved the application to perform the ex vivo skin infection study on rat skin (IRB # 01/02/2022–2023). After the collection of the skin samples, all fat and hair were removed from the surface of the skin, then the skin was cut into 2 × 2 cm pieces and sterilized by immersion in 70% alcohol. After that, the skin samples were dispensed on Muller-Hinton agar, and then aliquots of 50 µL of bacterial inoculum (10^3^–10^7^ CFU/mL) were applied and spread uniformly on the dry epidermal side of the skin using a sterile pipet tip. The plates were incubated at 37 °C for 2 h. Then, 25–100 µL of the treatments were applied on the infected side of each skin piece using a sterile pipet tip. Untreated skin was used as a positive control, whereas non-infected skin was used as a negative control. Then the plates containing the skin pieces were incubated at 37 °C for 24 h. After that, bacteria were collected from the skin piece using a sterile cotton swab dipped into normal saline. The swabbing procedure of the skin was performed 5–10 times. After the final swab, the cotton swab was placed in 1 mL of normal saline. The tube holding the cotton tip was vortexing in the solution, and the solution was squeezed out of the cotton tip into a sampling tube, and the swab was discarded. A one-log 10-fold serial dilution of the sampling solution was prepared in normal saline. Next, 100 μL of each dilution was applied and spread on nutrient agar using a sterile cotton swab, and the plates were incubated at 37 °C for 18–24 h. Finally, CFUs and log_10_ CFU/skin piece were calculated, and log_10_ CFU/site ± standard deviation (SD) was determined for each skin piece.

#### 4.2.13. Stability Studies of Bigels

##### Rheological Stability

The viscosity and viscoelastic properties of bigels were measured as described in Section 4.2.9 after storage for 3 months at room temperature to evaluate the rheological stability of bigels.

##### Physicochemical Stability

The thermodynamic stability of bigels was tested by the freezing/thawing cycle method as described [55]. Briefly, bigels were alternately frozen at −20 °C and thawed at 70 °C for 15 min. The experiment was repeated five times, with bigels visually examined after each cycle for phase separation. In addition, the short-term physical and chemical stability of bigels was assessed at 2–8 °C (refrigerated), 22 °C (room temperature), 30 °C/65% RH, and 40 °C/75% RH after 1, 2, and 3 months. For physical stability, bigels were evaluated for appearance, color, and phase separation. Whereas in chemical stability, bigels were evaluated by monitoring the change in pH and determining the CIP.HCl content in bigels as described in Section 4.2.8.

#### 4.2.14. Statistical Analysis

A one-way ANOVA followed by Tukey’s multiple comparisons was used to investigate the statistical significance of the droplet size of CIP.HCl NE between the initial size and those reported after 7, 14, 21, and 28 days. Moreover, a one-way ANOVA followed by Tukey’s multiple comparisons was performed to investigate the statistical significance of zones of inhibition of various formulations versus different bacterial strains and the Log10 colony-forming units per skin reduction obtained from an ex vivo skin infection model. All statistical analyses were performed using Prism-GraphPad 9.1 (GraphPad Software, Inc., San Diego, CA, USA) and were based on a *p* < 0.05 level of significance.

## Figures and Tables

**Table 1:** Droplet size, PDI, and ζ-potential of CIP.HCl NE as a function of time. The data are presented as mean ± SD (*n* = 3).

Time	Initial	7 Days	14 Days	21 Days	28 Days
Droplet size (nm)	17.27 ± 2.74	11.19 ± 0.11	22.88 ± 1.91	11.19 ± 0.01	17.73 ± 0.06
PDI	0.21 ± 0.01	0.40 ± 0.02	0.40 ± 0.01	0.48 ± 0.03	0.49 ± 0.01
ζ-potential (mV)	0.70 ± 0.01	0.80 ± 0.10	0.50 ± 0.01	0.61 ± 0.02	1.57 ± 0.23

**Table 2:** CIP.HCl content in CIP.HCL NEs after three months of storage at 2–8 °C, room temperature (22 °C), 30 °C/65% RH, and 40 °C/75% RH. The data are presented as mean ± SD (*n* = 3).

Time		2–8 °C	Room Temperature (22 °C)	30 °C/65% RH	40 °C/75% RH
	Storage Condition				
Initial (%)		100.6 ± 2.9	100.6 ± 2.9	100.6 ± 2.9	100.6 ± 2.9
1 month (%)		98.9 ± 1.6	100.8 ± 1.0	101.5 ± 1.3	100.3 ± 1.3
2 months (%)		96.5 ± 4.2	103.8 ± 2.7	101.2 ± 0.4	104.1 ± 3.6
3 months (%)		99.4 ± 1.7	102.1 ± 0.7	102.3 ± 0.3	105.6 ± 1.2

**Table 3:** Characteristic peaks in the FTIR spectra of F1–F5 bigels.

Bigels	O-H Stretching Vibration Peak (cm^−1^)	C=O Stretching Vibration Peak (cm^−1^)	C-O-C Stretching Peak (cm^−1^)	CH Stretching Vibration Peak (cm^−1^)
F1	3367	1652	1043	2853–2923
F2	3351	1645	1043	2853–2923
F3	3348	1642	1043	2854–2924
F4	3360	1642	1043	2854–2924
F5	3344	1636	1042	2854–2924

**Table 4:** Rheological parameters of the strain-sweep tests (LVR, applied strain within LVR, and critical strain (γ_C_)) of F1–F5 bigels.

Bigels	LVR (%)	Applied Strain within the LVR (%)	Critical Strain (γ_C_, %)
F1	0.016–5.500	0.10	5.500
F2	0.010–9.700	0.10	9.700
F3	0.010–10.000	0.10	10.000
F4	0.010–10.000	0.10	10.000
F5	0.010–1.000	0.10	1.000

**Table 5:** The kinetic parameters (n, K, and R^2^) of the Korsmeyer–Peppas model of F1–F5 bigels.

Bigels	K (h^−1^ )	n	R^2^
F1	16.73	1.07	0.9988
F2	19.96	1.01	0.9973
F3	18.05	1.09	0.9988
F4	19.02	1.03	0.9976
F5	23.47	1.12	0.9895

**Table 6:** Zones of inhibition of CIP.HCl NE and F1–F5 bigels against various bacterial strains by the well diffusion method. Data are presented as mean ± SD (*n* = 2).

Sample	Tested Sample	Gram-Negative Bacteria			Gram-Positive Bacteria	
		*B. subtilis* (mm*)*	*S. aureus* (mm)	*E. coli* (mm)	*P. mirabilis* (mm)	*P. aeruginosa* (mm)
1	0.3% Ciprocin eye/ear drops	43.5 ± 2.1 ***	37.5 ± 2.1 **	36.5 ± 2.1	38.5 ± 2.1 **	38.0 ± 0.0 **
2	1.5% CIP.HCl solution	49.5 ± 2.1 ***	42.0 ± 0.7 ***	40.5 ± 0.7	42.5 ± 0.7 ***	40.0 ± 0.0 ***
3	1.5% CIP.HCl NE	48.0 ± 0.0 ***	40.0 ± 0.0 **	36.0 ± 1.4	36.5 ± 0.7 **	41.0 ± 1.4 ***
3N	Blank NE	24.5 ± 2.1	14.0 ± 0.0	19.5 ± 0.7	23.0 ± 0.0	12.0 ± 0.0
4	F1	36.0 ± 0.0 **	28.5 ± 0.7 **	30.0 ± 2.8 **	29.0 ± 0.7 **	32.0 ± 0.0 **
4N	Blank of F1	8.0 ± 0.0	9.0 ± 0.0	NZ	NZ	NZ
5	F2	39.5 ± 0.7 **	29.5 ± 0.7 **	32.5 ± 0.7 **	34.0 ± 1.4 **	35.5 ± 2.1 **
5N	Blank of F2	7.0 ± 0.0	NZ	NZ	NZ	NZ
6	F3	40.0 ± 0.0 **	31.0 ± 0.0	32.0 ± 0.0	33.0 ± 0.7	34.0 ± 1.4
6N	Blank of F3	8.0 ± 0.0	NZ	NZ	NZ	NZ
7	F4	38.5 ± 0.0 **	29.0 ± 0.0	31.5 ± 2.8 *	31.0 ± 0.7 *	34.5 ± 0.7 **
7N	Blank of F4	9.0 ± 0.0	NZ	6.0 ± 0.0	NZ	NZ
8	F5	42.0 ± 0.7 **	33.5 ± 0.7 **	35.0 ± 0.7 **	36.0 ± 0.7 **	36.5 ± 2.8 **
8N	Blank of F5	9.0 ± 0.0	7.0 ± 0.0	NZ	NZ	NZ

NZ: no zone; Wells (1 and 2): positive controls; Wells (3, 4, 5, 6, 7, and 8): CIP.HCl NE and F1–F5; and Wells (3N, 4N, 5N, 6N, 7N, and 8N): blank samples of NE and F1–F5. * *p* < 0.05, ** *p* < 0.01, *** *p* < 0.001 compared to Blank NE.

**Table 7:** The antimicrobial activity of F3 and F5 against Gram-positive and Gram-negative bacteria.

Treatment	Gram-Negative Bacteria						Gram-Positive Bacteria			
	*E. coli*		*P. aeruginosa*		*P. mirabilis*		*B. subtilis*		*S. aureus*	
	Mean log_10_ CFU/Skin ± SD after 24 h Post-Treatment	Log_10_ CFU/Skin Reduction vs. Control	Mean log_10_ CFU/Skin ± SD after 24 h Post-Treatment	Log_10_ CFU/Skin Reduction vs. Control	Mean log_10_ CFU/Skin ± SD after 24 h Post-Treatment	Log_10_ CFU/Skin Reduction vs. Control	Mean log_10_ CFU/Skin ± SD after 24 h Post-Treatment	Log_10_ CFU/Skin Reduction vs. Control	Mean log_10_ CFU/skin ± SD after 24 h Post-Treatment	Log_10_ CFU/Skin Reduction vs. Control
No treatment (positive control)	9.74 ± 1.75	N/A	9.63 ± 1.64	N/A	9.55 ± 1.76	N/A	9.76 ± 1.78	N/A	9.58 ± 1.96	N/A
Treatment 1 (F3)	2.70 ± 0.02	−7.04 *	2.30 ± 0.02	−7.33 *	NC	−9.55 *	4.41 ± 0.02	−5.35 *	NC	−9.58 *
Treatment 2 (F5)	NC	−9.74 *	NC	−9.63 *	NC	−9.55 *	NC	−9.76 *^,#^	NC	−9.58 *
Negative control	NC	N/A	NC	N/A	NC	N/A	NC	N/A	NC	N/A

NC: no colony; N/A: not applicable; ∗ *p* < 0.001 (F3 and F5 vs. positive control); and # *p* < 0.0001 (F5 vs. F3).

**Table 8:** pH of F2–F5 bigels stored at 2–8 °C, room temperature (22 °C), 30 °C/65% RH, and 40 °C/75% RH for three months. The data are presented as mean ± SD (*n* = 3).

Bigels	Initial	One Month				Two Months				Three Months			
		2–8 °C	22 °C	30 °C/ 65% RH	40 °C/ 75% RH	2–8 °C	22 °C	30 °C/ 65% RH	40 °C/ 75% RH	2–8 °C	22 °C	30 °C/ 65% RH	40 °C/ 75% RH
F2	6.5 ± 0.1	6.4 ± 0.0	6.0 ± 0.0	6.0 ± 0.1	6.0 ± 0.3	6.1 ± 0.2	6.1 ± 0.1	6.1 ± 0.1	6.1 ± 0.3	6.2 ± 0.2	6.1 ± 0.0	6.1 ± 0.0	6.2 ± 0.2
F3	6.5 ± 0.2	6.0 ± 0.1	5.9 ± 0.0	5.9 ± 0.2	5.9 ± 0.0	5.9 ± 0.1	5.9 ± 0.0	6.0 ± 0.2	5.8 ± 0.0	5.9 ± 0.1	5.9 ± 0.0	5.9 ± 0.1	5.9 ± 0.0
F4	6.5 ± 0.0	5.9 ± 0.0	6.1 ± 0.0	6.0 ± 0.0	6.2 ± 0.0	6.3 ± 0.0	6.2 ± 0.2	6.1 ± 0.0	6.1 ± 0.1	6.1 ± 0.0	6.3 ± 0.2	6.1 ± 0.2	6.1 ± 0.1
F5	6.5 ± 0.0	5.9 ± 0.0	5.9 ± 0.2	5.9 ± 0.0	5.7 ± 0.1	6.1 ± 0.0	6.0 ± 0.2	6.0 ± 0.2	6.0 ± 0.2	6.0 ± 0.1	6.0 ± 0.1	5.9 ± 0.0	6.1 ± 0.1

**Table 9:** CIP.HCl content in bigels stored at 2–8 °C, room temperature (22 °C), 30 °C/65% RH, and 40 °C/75% RH for three months. The data are presented as mean ± SD (*n* = 3).

Bigels	Initial	One Month				Two Months				Three Months			
		2–8 °C	22 °C	30 °C/ 65% RH	40 °C/ 75% RH	2–8 °C	22 °C	30 °C/ 65% RH	40 °C/ 75% RH	2–8 °C	22 °C	30 °C/ 65% RH	40 °C/ 75% RH
F2	92.1 ± 1.1	85.6 ± 4.0	86.8 ± 3.3	82.2 ± 2.5	80.8 ± 4.2	85.2 ± 2.6	84.3 ± 3.5	82.3 ± 2.0	79.2 ± 3.7	82.8 ± 4.1	83.3 ± 3.6	80.1 ± 2.6	77.2 ± 3.8
F3	93.6 ± 2.9	91.5 ± 2.1	88.6 ± 1.4	86.4 ± 0.7	85.0 ± 4.9	90.5 ± 4.0	88.7 ± 2.0	86.0 ± 3.2	84.6 ± 0.5	90.7 ± 0.5	86.7 ± 2.9	85.7 ± 3.2	83.9 ± 5.7
F4	93.6 ± 4.3	88.6 ± 2.3	88.6 ± 3.2	84.1 ± 1.2	82.5 ± 3.3	90.4 ± 3.0	87.3 ± 2.6	83.4 ± 4.3	81.4 ± 1.9	87.4 ± 2.7	85.1 ± 4.8	80.6 ± 3.7	80.3 ± 2.1
F5	94.1 ± 2.6	91.2 ± 2.4	91.1 ± 2.3	92.3 ± 1.4	90.6 ± 3.1	90.9 ± 0.2	91.9 ± 5.0	91.4 ± 5.2	91.0 ± 3.2	90.4 ± 3.3	92.2 ± 3.6	92.4 ± 3.2	89.8 ± 2.3

**Table 10:** Composition of Type I bigels prepared with different ratios of OL:WH.

Type I Bigels	OL:WH Ratio	CIP.HCl NE (mL)	WH Phase (g/mL)		OL Phase (g/mL)		The Phase in Which CIP.HCl NE Was Dispersed
			CP (g)	DW (mL)	EC (g)	OA (mL)	
F1	1:1	20	0.20	40.0	4.44	40.0	OL
F2	1:2	20	0.27	53.3	2.97	26.7	OL
F3	1:4	20	0.32	64.0	1.78	16.0	OL
F4	1:2	20	0.27	53.3	2.97	26.7	WH
F5	1:4	20	0.32	64.0	1.78	16.0	WH

**Table 11:** Composition of Type II bigels prepared with different ratios of WH:OL.

Type II Bigels	WH:OL Ratio	CIP.HCl NE (mL)	WH Phase (g/mL)		OL Phase (g/mL)		The Phase in Which CIP.HCl NE Was Dispersed
			CP (g)	DW (mL)	EC (g)	OA (mL)	
F6	1:1	20	0.20	40	4.44	40	WH
F7	1:2	20	0.13	26.7	5.92	53.3	WH
F8	1:4	20	0.08	16.0	7.11	64.0	WH
F9	1:2	20	0.13	26.7	5.92	53.3	OL
F10	1:4	20	0.08	16.0	7.11	64.0	OL

## Data Availability

The data presented in this study are available upon request from the corresponding author.

## Supplementary Materials

The following supporting information can be downloaded at: https://www.mdpi.com/article/10.3390/gels9070592/s1, Figure S1. A clear and homogenous CIP.HCl NE; Figure S2. Crystallization of CIP.HCl NE at very low temperature (−20 ºC) during a freezing/thawing cycles; Figure S3. *In vitro* antimicrobial activity of CIP.HCl NE and F1–F5 against: (A) *B. subtilis,* (B) *S. aureus,* (C) *E. coli,* (D) *P. mirabilis, and* (E) *P. aeruginosa.* Wells (1 and 2): Positive controls, Wells (3, 4, 5, 6, 7, and 8): CIP.HCl NE and F1–F5 bigels, and Wells (3N, 4N, 5N, 6N, 7N, and 8N): Blank samples of NE and F1–F5; Figure S4. *Ex vivo* antimicrobial activity by the skin infection model of F3 and F5 bigels against: (A) *E. coli*, (B) *P. aeruginosa*, (C) *P. mirabilis*, (D) *B. subtilis*, and (E) *S. aureus.* Treatment 1: F3 and Treatment 2: F5; Figure S5. A schematic diagram of the preparation of Type I bigels; Figure S6. A schematic diagram of the preparation of Type II bigels.
