# Supplementary figures and images for: Development and Comparative Evaluation of Ciprofloxacin Nanoemulsion-Loaded Bigels Prepared Using Different Ratios of Oleogel to Hydrogels
Source: Gels. 2023 Jul 23;9(7):592. doi: 10.3390/gels9070592 (PMC10379317; doi:10.3390/gels9070592)

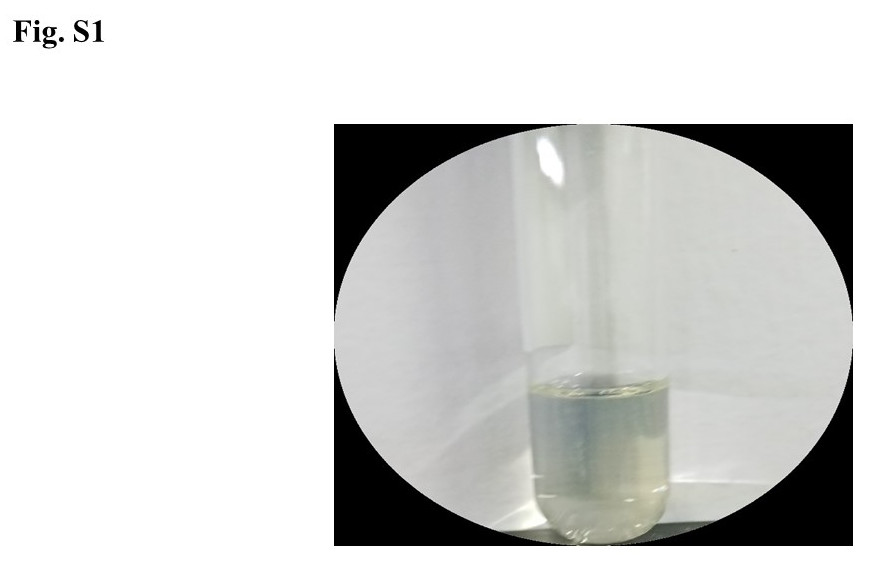

Supplement: Supplementary file 1 [file gels-09-00592-s001.zip › FIG_S1.jpg]

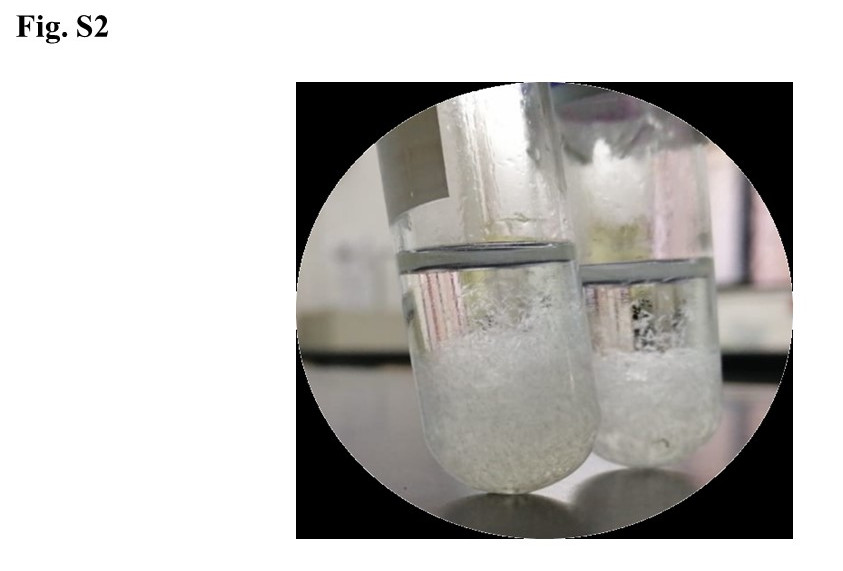

Supplement: Supplementary file 1 [file gels-09-00592-s001.zip › FIG_S2.jpg]

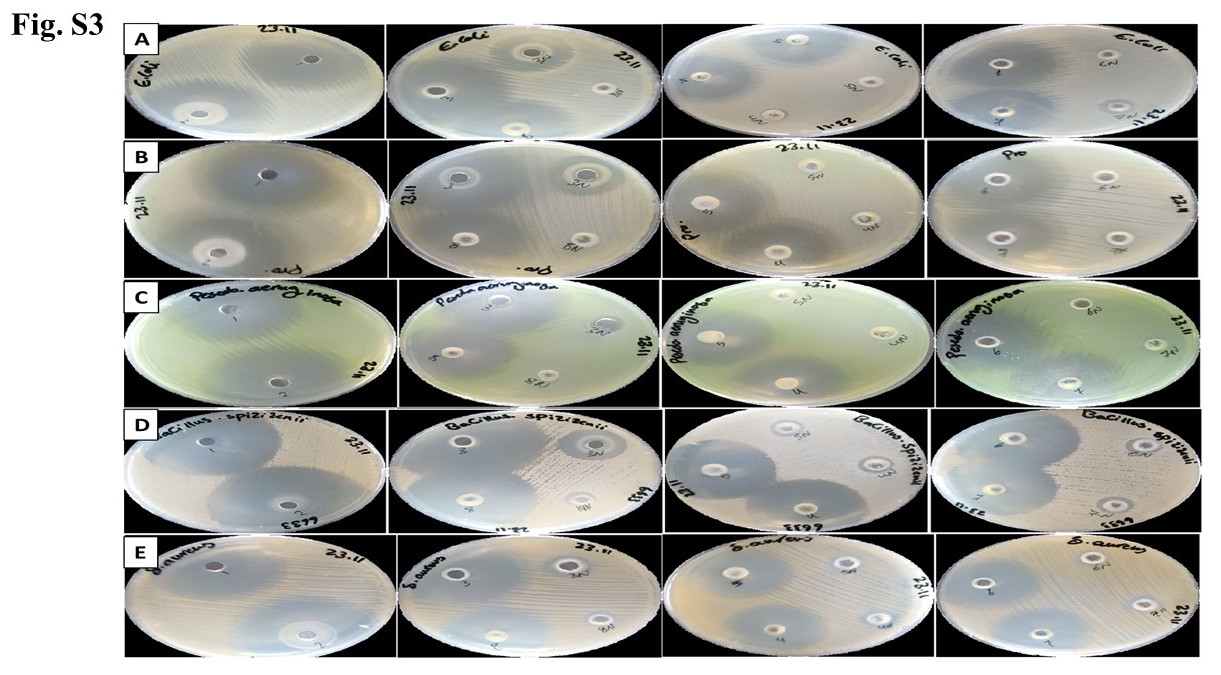

Supplement: Supplementary file 1 [file gels-09-00592-s001.zip › FIG_S3.jpg]

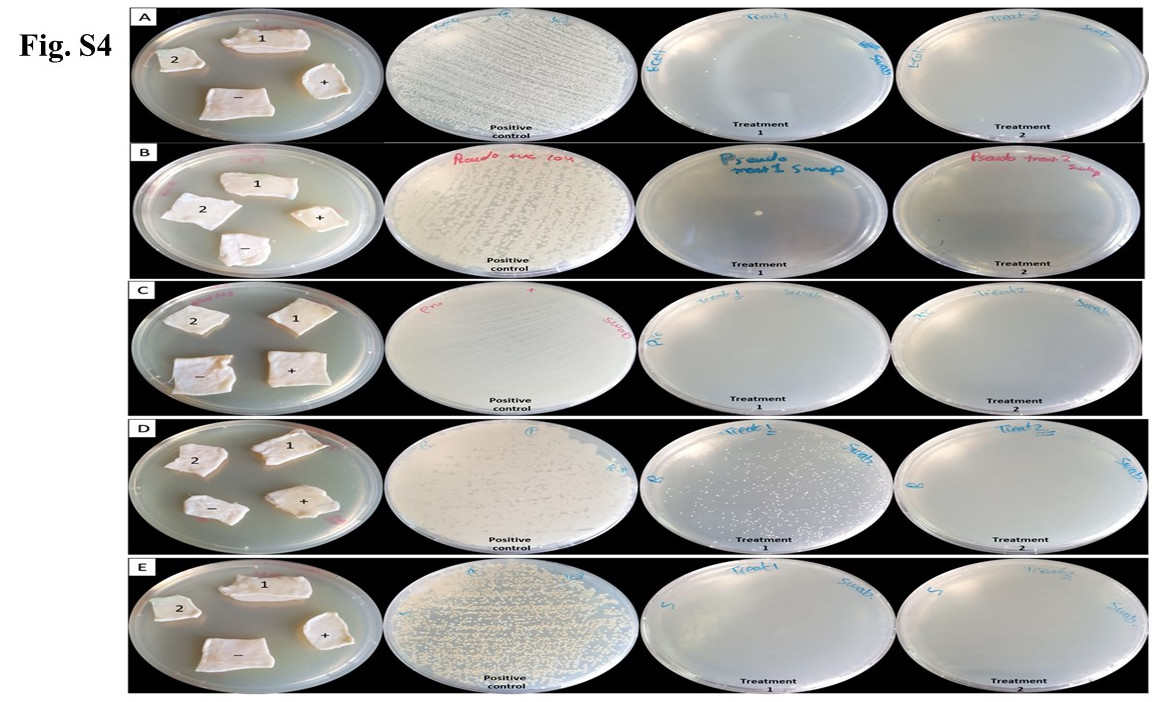

Supplement: Supplementary file 1 [file gels-09-00592-s001.zip › FIG_S4.jpg]

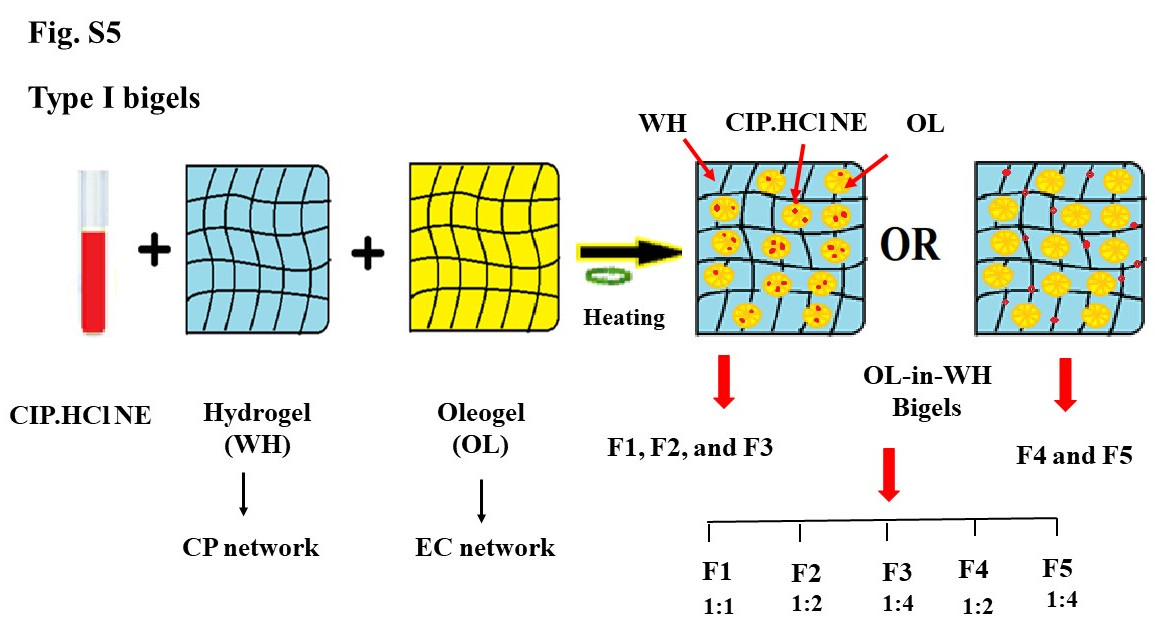

Supplement: Supplementary file 1 [file gels-09-00592-s001.zip › FIG_S5.jpg]

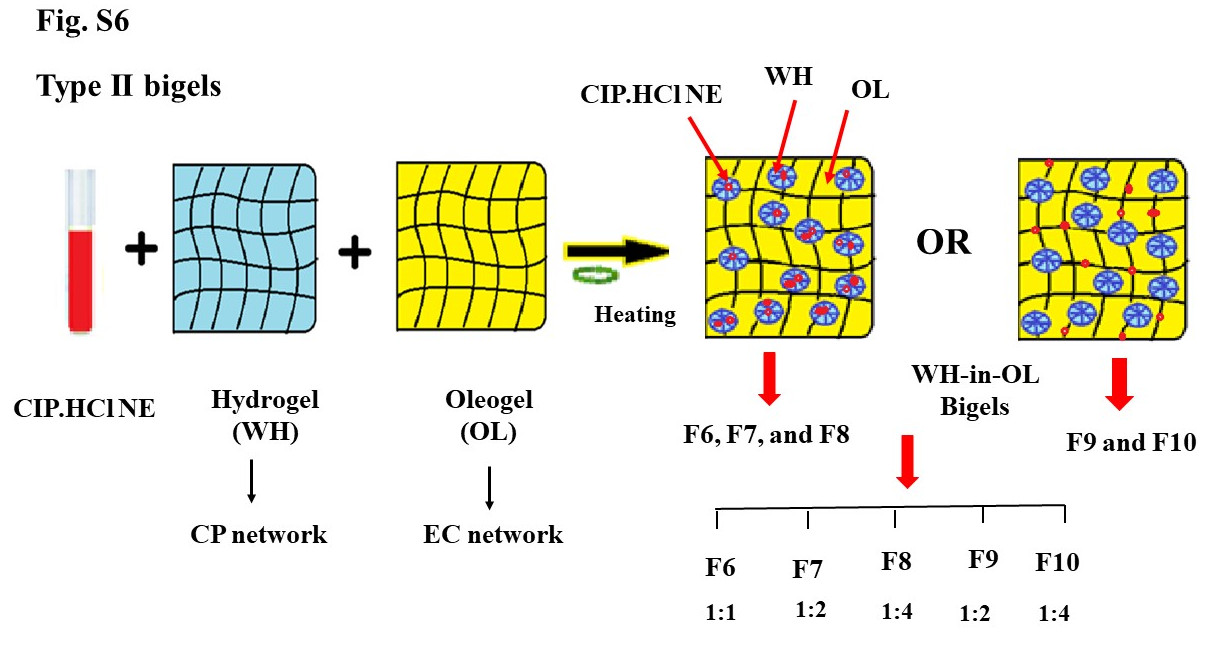

Supplement: Supplementary file 1 [file gels-09-00592-s001.zip › FIG_S6.jpg]
